# Peer Review of “Health Care System Overstretch and In-Hospital Mortality of Intubated Patients With COVID-19 in Greece From September 2020 to April 2022: Updated Retrospective Cohort Study”

**DOI:** 10.2196/59638

**Published:** 2024-06-10

**Authors:** Mario Coccia

**Affiliations:** 1 National Research Council of Italy, Turin, Italy

**Keywords:** COVID-19, pandemic, health care disparities, intensive care unit, ICU, right to health, quality of care, intubation, mortality, health disparity, health inequality, surveillance data, in-patient, mortality, COVID-19 patient, hospitalization, disparity, inequality, surveillance, health care system, Greece, region, Delta, Omicron, vaccination, vaccine, public health, patient load, deterioration, time

*This is the peer-review report for “Health Care System Overstretch and In-Hospital Mortality of Intubated Patients With COVID-19 in Greece From September 2020 to April 2022: Updated Retrospective Cohort Study.”*

## Round 1 Review

### General and Detailed Comments

The topics of this paper [1] are interesting, though well known.

The structure and content must be revised, and results have to be better explained by authors before being reconsidered for publication.

Title has to be shorter.

Abstract has to clarify the goal, sample, results, and health and social implications to cope with the next pandemics to improve health care.

Introduction is poor and has to better clarify the research questions of this study and provide more theoretical background about strategies of prevention and good governance to cope with the pandemic crisis. After that they can focus on the topics of this study to provide a correct analysis for fruitful discussion (see suggested readings that must be all read and used in the text).

Methods of this study are not clear. The section of Materials and Methods must be restructured with the following 3 points in the same order:

- Sample and data
- Measures of variables
- Models and data analysis procedure

Results: It is not clear why authors apply a Mann-Whitney test, considering the large sample it is better to apply an independent sample *t* test, or some other parametric test. This additional test can support better results if they are reliable. Table 1: avoid acronyms; specify ICU as intensive care unit. Same comments for Figure 2.

Discussion: first, authors have to synthesize the main results in a simple table to be clear for readers and then show what this study adds compared to other studies. In addition, authors should discuss the type of mechanical ventilation, because studies show that invasive ventilation (intubation) creates VAP (ventilator-associated pneumonia), and a lot of people intubated for COVID-19 died from this problem rather than COVID-19. Countries that have reduced mortality, such as Germany and New Zealand, used mainly noninvasive ventilation, which can better treat patients and avoid mortality with new technology. See suggested papers.

Conclusion has to be added as an autonomous section. Conclusion not to be a summary, but authors have to focus on manifold limitations of this study and provide suggestions for health, crisis management, and social policy, as well as how nations can prevent, with good governance and new technology in artificial ventilation, the next pandemics and improve health care with vaccination, noninvasive ventilation, and nonpharmaceutical measures of control. In this manner the paper can provide useful policy implications for Greece and improve health care for the next pandemics.

Overall, then, the paper is interesting. The theoretical framework is weak, and some results create confusion...the structure of the paper has to be improved; study design, discussion, and presentation of results have to be clarified using the suggested comments.

I strongly suggest improving the paper by using all comments (suggested papers that are included to all be read and used) that I will verify in-depth, and maybe it can be considered. If the paper is not improved as suggested it will be dismissed.

Suggested readings of relevant papers that have to be read and all inserted in the text and references:

Nasrullah A, Gangu K, Garg I, et al. Trends in hospitalization and mortality for influenza and other respiratory viruses during the COVID-19 pandemic in the United States. Vaccines (Basel). Feb 10, 2023;11(2):412.

Coccia M. High potential of technology to face new respiratory viruses: mechanical ventilation devices for effective healthcare to next pandemic emergencies. Technol Soc. May 2023;73:102233.

COVID-19 Forecasting Team. Variation in the COVID-19 infection–fatality ratio by age, time, and geography during the pre-vaccine era: a systematic analysis. Lancet. Apr 16, 2022;399(10334):1469-1488.

Benati I, Coccia M. Effective contact tracing system minimizes COVID-19 related infections and deaths: policy lessons to reduce the impact of future pandemic diseases. J Pub Admin Gov. Sept 2022;12(3):19-33.

Velissaris D, Paraskevas T, Oikonomou E, Bizos A, Karamouzos V, Marangos M. Evaluation of four novel prognostic scores on admission for COVID-19 mortality. An experience from a Mediterranean tertiary center. Acta Clin Belg. Aug 2022;77(4):748-752.

Magazzino C, Mele M, Coccia M. A machine learning algorithm to analyse the effects of vaccination on COVID-19 mortality. Epidemiol Infect. Sept 12, 2022;150:e168.

Kondilis E, Tarantilis F, Benos A. Essential public healthcare services utilization and excess non-COVID-19 mortality in Greece. Public Health. Sep 2021;198:85-88.

Coccia M. Sources, diffusion and prediction in COVID-19 pandemic: lessons learned to face next health emergency. AIMS Public Health. Mar 2, 2023;10(1):145-168.

Mazzucchelli R, Agudo Dieguez A, Dieguez Costa EM, Crespí Villarías N. Democracia y mortalidad por COVID-19 en Europa. Rev Esp Salud Publica. Jun 24, 2020;94:e202006073.

Coccia M. Preparedness of countries to face COVID-19 pandemic crisis: strategic positioning and underlying structural factors to support strategies of prevention of pandemic threats. Environ Res. Jan 2022;203:111678.

Marinaki C, Kapadochos T, Katsoulas T, et al. Estimation of the optimal time needed for weaning of intensive care unit tracheostomized patients on mechanical ventilation. A prospective observational study. Acta Biomed. Apr 24, 2023;94(2):e2023103.

Coccia M. Optimal levels of vaccination to reduce COVID-19 infected individuals and deaths: a global analysis. Environ Res. Mar 2022;204(Pt C):112314.

Maltezou HC, Basoulis D, Bonelis K, et al. Effectiveness of full (booster) COVID-19 vaccination against severe outcomes and work absenteeism in hospitalized patients with COVID-19 during the Delta and Omicron waves in Greece. Vaccine. Mar 31, 2023;41(14):2343-2348.

Benati I, Coccia M. Global analysis of timely COVID-19 vaccinations: improving governance to reinforce response policies for pandemic crises. Int J Health Governance. Aug 12, 2022;27(3):240-253.

Stasinos N, Kousis A, Sarlis V, et al. A tri-model prediction approach for COVID-19 ICU bed occupancy: a case study. Algorithms. 2023;16(3):140.

Coccia M. Comparative critical decisions in management. In: Farazmand A, editor. Global Encyclopedia of Public Administration, Public Policy, and Governance. Springer Nature. 2020.

Aslanidis V, Tsolaki V, Papadonta ME, et al. The impact of the COVID-19 pandemic on mental health and quality of life in COVID-19 department healthcare workers in Central Greece. J Pers Med. Jan 29, 2023;13(2):250.

Coccia M. Factors determining the diffusion of COVID-19 and suggested strategy to prevent future accelerated viral infectivity similar to COVID. Sci Total Environ. Aug 10, 2020;729:138474.

Panagiotopoulos ΦI. Corporate social responsibility initiatives and programs in the health system of Greece due to the pandemic of COVID-19. In: Indowu MT, Idowu AO, editors. Corporate Social Responsibility in the Health Sector. CSR, Sustainability, Ethics & Governance. Springer. 2023:93-110.

Coccia M. COVID-19 pandemic over 2020 (with lock downs) and 2021 (with vaccinations): similar effects for seasonality and environmental factors. Environ Res. May 15, 2022;208:112711.

Malli F, Lampropoulos IC, Perlepe G, Papagiannis D, Gourgoulianis KI. Analysis of SARS-CoV-2 cases, COVID-19 outcomes and vaccinations, during the different SARS-CoV-2 variants in Greece. Vaccines (Basel). Jan 4, 2023;11(1):126.

Coccia M. Pandemic prevention: lessons from COVID-19. Encyclopedia. 2021;1(2):433-444.

Gavrielatou E, Vaporidi K, Tsolaki V, et al. Rapidly improving acute respiratory distress syndrome in COVID-19: a multi-centre observational study. Respir Res. Apr 14, 2022;23(1):94.

Coccia M. Effects of strict containment policies on COVID-19 pandemic crisis: lessons to cope with next pandemic impacts. Environ Sci Pollut Res Int. Jan 2023;30(1):2020-2028.

Stafylaki D, Maraki S, Vaporidi K, et al. Impact of molecular syndromic diagnosis of severe pneumonia in the management of critically ill patients. Microbiol Spectr. Oct 26, 2022;10(5):e0161622.

Coccia M. Covid-19 vaccination is not a sufficient public policy to face crisis management of next pandemic threats. Public Organiz Rev. Oct 17, 2022;23:1353-1367.

Rapti I, Asimakopoulos A, Liontos A, et al. Association of patient characteristics with clinical outcomes in a cohort of hospitalised patients with SARS-CoV-2 infection in a Greek referral centre for COVID-19. Epidemiol Infect. Aug 16, 2022;150:e160.

Coccia M. Improving preparedness for next pandemics: max level of COVID-19 vaccinations without social impositions to design effective health policy and avoid flawed democracies. Environ Res. Oct 2022;213:113566.

Lytras T, Tsiodras S. Total patient load, regional disparities and in-hospital mortality of intubated COVID-19 patients in Greece, from September 2020 to May 2021. Scand J Public Health. Aug 2022;50(6):671-675.
